# Molecular Requirements for Peroxisomal Targeting of Alanine-Glyoxylate Aminotransferase as an Essential Determinant in Primary Hyperoxaluria Type 1

**DOI:** 10.1371/journal.pbio.1001309

**Published:** 2012-04-17

**Authors:** Krisztián Fodor, Janina Wolf, Ralf Erdmann, Wolfgang Schliebs, Matthias Wilmanns

**Affiliations:** 1 European Molecular Biology Laboratory Hamburg, Hamburg, Germany; 2 Department of Systems Biology, Faculty of Medicine, Institute for Physiological Chemistry, Ruhr University of Bochum, Bochum, Germany; Princeton University, United States of America

## Abstract

Alanine-glyoxylate aminotransferase is a peroxisomal enzyme, of which various missense mutations lead to irreversible kidney damage via primary hyperoxaluria type 1, in part caused by improper peroxisomal targeting. To unravel the molecular mechanism of its recognition by the peroxisomal receptor Pex5p, we have determined the crystal structure of the respective cargo–receptor complex. It shows an extensive protein/protein interface, with contributions from residues of the peroxisomal targeting signal 1 and additional loops of the C-terminal domain of the cargo. Sequence segments that are crucial for receptor recognition and hydrophobic core interactions within alanine-glyoxylate aminotransferase are overlapping, explaining why receptor recognition highly depends on a properly folded protein. We subsequently characterized several enzyme variants in vitro and in vivo and show that even minor protein fold perturbations are sufficient to impair Pex5p receptor recognition. We discuss how the knowledge of the molecular parameters for alanine-glyoxylate aminotransferase required for peroxisomal translocation could become useful for improved hyperoxaluria type 1 treatment.

## Abstract 2

The crystal structure of the peroxisome enzyme alanine-glyoxylate aminotransferase bound to its targeting receptor Pex5p explains why even minor fold defects prevent targeting of the enzyme and cause kidney disease.

## Author Summary

Peroxisomes are cell organelles contain proteins involved in various aspects of metabolism. Peroxisome proteins translocate from their site of synthesis in the cytoplasm across the organelle membrane in a fully folded and functional form. One such protein is the enzyme alanine–glyoxylate aminotransferase (AGT). It contains a targeting signal in its C-terminus that is recognized by a receptor protein, Pex5p, in the cytoplasm, which allows its subsequent translocation into the peroxisome. Mutations in AGT cause a disease known as primary hyperoxaluria type 1, in which patients suffer irreversible kidney damage; this disease results, in many cases, from improper targeting of AGT into peroxisomes. To understand better the mechanism of AGT import into peroxisomes and the molecular basis of this disease, we have determined the crystal structure of the complex between AGT and its receptor Pex5p. The structure reveals how overlapping segments of the protein sequence are crucial for both receptor recognition and maintaining the folded structure of the enzyme. Subsequently, we created and studied several mutants of the enzyme, including mutants that are known to cause disease, and found that even minor folding defects in the enzyme prevent its recognition by Pexp5 and its import into peroxisomes. Our data thus provide novel insights into the consequences of mutations in AGT on the catalytic activity of the enzyme, as well as into the mechanisms that cause primary hyperoxaluria type 1.

## Introduction

Primary hyperoxaluria type 1 (PH1) is an autosomal recessive disorder that generally becomes symptomatic during childhood or adolescence and ultimately leads to renal failure, usually between the ages of 25 and 45 [1]. Although several therapeutic options have been established, the only curative treatment to date is by liver-kidney transplantation [2]. At the molecular level, PH1 is caused by functional deficiencies in the liver-specific, pyridoxal-dependent enzyme alanine-glyoxylate aminotransferase (AGT, EC 2.6.1.44) [3]. AGT catalyzes the transamination of the peroxisomal intermediary metabolite glyoxylate to glycin. Human AGT consists of a 86 kDa homodimer and bears an atypical Lys-Lys-Leu (KKL) peroxisomal targeting signal 1 (PTS1) motif at its C-terminus, which is required for translocation of the enzyme into peroxisomes. The absence of AGT in hepatic peroxisomes, owing to either dysfunction or mistargeting of AGT, causes glyoxylate to escape into the cytosol where it is further metabolized to oxalate and glycolate. The accumulation of oxalate—a compound that cannot be further metabolized in humans—leads to the progressive formation of insoluble calcium oxalate in the kidney and urinary tract, resulting in urolithiasis and/or nephrocalcinosis as the principal clinical manifestations.

To date, around 150 polymorphic variants of the human *AGXT* gene have been described [4]. These mutations are scattered over virtually the entire encoded AGT sequence and the associated three-dimensional structure of the enzyme (Figure S1). In 2%–20% of human populations in geographically distinct regions, a minor allele haplotype (*AGXT-Mi*) is found, which encodes an AGT variant with two missense mutations (P11L, I340M). AGT-Mi has around one-third of the catalytic activity of the wild-type enzyme and reduced stability, yet by itself does not lead to a serious clinical phenotype. However, the presence of *AGXT-Mi* in combination with further mutations causes almost 50% of the reported PH1, demonstrating synergistic disease effects [4]. Only some of the characterized PH1-causing *AGXT* variants can be directly correlated with AGT enzymatic activity, suggesting that other molecular parameters such as its correct compartmental localization have important implications for AGT function as well. Therefore, it is not surprising that there is no uniform response by PH1 patients to pyridoxine intake, which is thought to stabilize the AGT active site but does not directly affect the localization of the enzyme [5],[6].

On the basis of biochemical and structural data, the molecular mechanism of AGT catalytic activity is well established [7],[8], but the mechanism of peroxisomal AGT targeting is poorly understood. The non-canonical PTS1 Lys-Lys-Leu sequence in human AGT has been described as non-optimal, based on in vitro interaction studies of chimeric proteins formed by fusing the motif with non-human AGTs and other peroxisomal target proteins [7],[9]. Truncation studies of human AGT led to the prediction of an additional binding site within the small C-terminal domain of AGT, proximal to the established PTS1 C-terminus [9]. Another non-overlapping AGT-Pex5p recognition segment was proposed to be located close to the AGT N-terminus [10]. However, in the absence of residue-specific interaction data, it is not known whether additional interactions with the Pex5p receptor are direct or mediated by putative adaptors, or even whether allosteric effects are involved [3],[11].

Moreover, a generalization of the interpretation of available data is virtually impossible, as neither the PTS1 sequence nor a consistent pattern for peroxisomal localization are taxonomically conserved among AGTs from different species [12]. Indeed, depending on the organism, AGTs have been found, partly in parallel, in mitochondria, the cytosol, and peroxisomes [13]. Alternative transcription and translation sites in several AGTs lead to elongated isoforms with an additional N-terminal mitochondrial targeting signal sequence, which overrides the PTS1 required for peroxisomal translocation [3],[14]. Even in the absence of an additional mitochondrial-targeting signal, residual mitochondrial localization has been observed for AGT mutants that tend to aggregate and misfold [4],[14].

The aim of this work has been to unravel the role of non-PTS1 PH1 mutations in AGT mistargeting, to ultimately provide a molecular model for genetically imprinted PH treatment. To identify the complete Pex5p receptor-interaction site, we have first determined the atomic structure of the AGT–Pex5p receptor complex, which forms an elongated Pex5p-(AGT)_2_-Pex5p assembly. In addition to the established PTS1-binding site, the structure reveals extensive but rather non-specific contributions from sequence segments of the C-terminal AGT domain. To test how perturbations in the AGT structure could result in effects on AGT–Pex5p receptor binding, we mutated several residues of the AGT C-terminal domain near the Pex5p interface and investigated the properties of the resulting mutants by biophysical and functional in vitro and in vivo assays, as well as their ability to bind Pex5p. The interactions observed are highly sensitive to any minor changes in the AGT structure caused by single-residue mutations—including those that have been identified in PH1 patients—demonstrating that non-PTS1 interactions are essential in Pex5p receptor recognition.

## Results

### Overall Structure of the Pex5p-(AGT)_2_-Pex5p Receptor–Cargo Complex

To determine the molecular basis of the recognition of AGT by the peroxisomal import receptor Pex5p and its implications in PH1, we purified human AGT and the C-terminal cargo-binding segment of human Pex5p (residues 315–639), referred to as Pex5p(C) [15]. The AGT–Pex5p(C) complex forms with an apparent (1∶1) stoichiometry and has a moderate dissociation constant of 3.5 µM (Table 1). AGT, alone or in complex with Pex5p(C), has a catalytic activity of close to 2,000 µM mg^−1^ h^−1^, which is in agreement with previously reported AGT data [16],[17] and suggests that binding to Pex5p does not compromise AGT activity.

**Table 1:** Functional characterization of AGT variants.

	Activity (µmol mg^−1^ h^−1^)			AGT Localization (%)^a^
AGT Variant	−Pex5p	+Pex5p	Pex5p Binding (µM)	Peroxisomal
Wild-type	1,975±336	2,020±238	3.5±0.4	93.3
Y330W	2,060±263	2,052±179	7.9±1.0	66.7
Y330A	1,932±279	2,073±230	19.4±8.3	59.7
A328W	1,822±318	2,025±160	6.2±0.8	69.3
V376D^b^	—	—	—	1.7
L380D^b^	—	—	—	0.0
V376P^b^	—	—	—	0.3
L380P^b^	—	—	—	0.0
V336D	1,700±190	1,775±169	3.5±0.2	75.7
G170R	1,319±211	1,396±174	3.8±0.7	76.0
V336D/G170R^b^	—	—	—	28.0

a For further details and standard deviations, see Figure 3.

b Soluble protein could not be obtained from heterologous protein expression.

**Figure 1:**
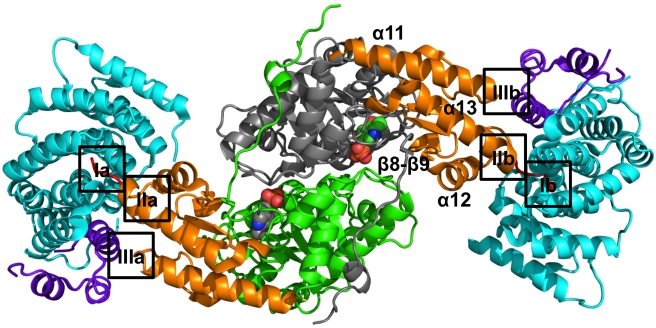
Overall structure of the Pex5p(C)–AGT_2_–Pex5p(C) complex. Each of the two Pex5p(C) molecules is colored in cyan (TPR domains) and violet (C-terminal helical bundle). The two AGT molecules are colored in green/grey (large catalytic domain), orange (C-terminal domain), and red (PTS1 segment). The secondary structural elements of the C-terminal domain of one AGT protomer are labeled. Two PLP molecules, bound to each AGT protomer, are shown in sphere presentation with atom-specific colors. The AGT-Pex5p binding interface can be subdivided into three surface patches, labeled I–III. The specific interactions found in these interfaces are shown in Figure S5. A zoom of the complete interface is shown in Figure 2A.

We then determined the crystal structure of the AGT–Pex5p(C) complex at 2.4 Å resolution (Figures 1 and S2; Table 2; Text S1). The structure comprises an elongated Pex5p(C)-(AGT)_2_-Pex5p(C) assembly with overall dimensions of around 140 Å×50 Å×50 Å. The 1∶2∶1 stoichiometry of the complex is in agreement with our isothermal titration microcalorimetry (ITC), gel filtration, and static light scattering data, indicating equal stoichiometric contributions of both protein components (Table S1 and Figure S3).

**Table 2:** X-ray structure determination.

	Pex5p(C)-AGT
**Data collection**	
Space group	P2_1_
Cell dimensions	
*a*, *b*, *c* (Å)	59.4, 99.2,127.7
α, β, γ(°)	90.0, 96.7, 90.0
Resolution (Å)	78.1–2.35 (2.48–2.35)
*R*_merge_	11.5 (60.9)
*I*/σ*I*	10.5 (2.0)
Completeness (%)	99.7 (97.9)
Redundancy	3.7 (3.4)
**Refinement**	
Resolution (Å)	78.1–2.35
Number of reflections	57,997
*R*_work_/*R*_free_	18.7/23.9
Number of atoms	
Protein	10,545
LYS-PLP	46
Water	831
*B*-factors	
Protein	31.9
LYS-PLP	34.2
Water	31.5
R.m.s. deviations	
Bond lengths (Å)	0.008
Bond angles (°)	1.082

Each of the two complete AGT polypeptide chains is visible in the final electron density, except for N-terminal residues 1–3 and 1–5, respectively. The overall conformation of the two AGT molecules is identical (Table S2) and the structure shows that they both contain the cofactor pyridoxal-5′-phosphate (PLP) covalently bound to Lys209 (Figure S4). We confirmed the AGT PLP-adduct to be present by spectroscopic analysis of the protein material submitted for crystallization (Figure S4C). The Pex5p(C)-bound AGT dimer superimposes well onto that of the enzyme in the absence of the receptor (PDB entry 1HOC) [8], with a root-mean-squares deviation of 0.41 Å (Table S2). This confirms that AGT dimeric assembly and overall conformation, a prerequisite for AGT catalytic activity [3], is not affected by Pex5p receptor binding.

Well interpretable electron density is visible for most of the two Pex5p(C) receptor molecules (residues 315–639), with the exception of the N-termini (residues 315–323/324), part of the distorted tetratricopeptide repeat (TPR) 4 segment (residues 441–464, 444–460) and the so-called 7C-loop (residues 591–592, 590–596) that connects the 7-fold array of TPR segments with the C-terminal bundle of Pex5p(C) [15]. These regions were either invisible or mobile in previous structures of the same receptor [15],[18], indicating that these sequence segments are generally flexible. Overall, increased flexibility of Pex5p(C), which we attribute to these regions and to the loose arrangement of neighboring TPR domain modules, is reflected in higher root-mean-squares deviations of around 1 Å when Pex5p(C) polypeptide chains of the Pex5p(C)-AGT complex are either superimposed on each other or onto the coordinates of the same receptor from the previously determined Pex5(C)-SCP2 cargo complex (Table S2) [15].

By contrast, there are significant deviations in the overall structure of Pex5p(C) bound to AGT when it is superimposed onto the apo conformation of the same receptor (PDB entry 2C0M). The matching part of the respective structures is limited to residues of the 7-fold TPR array, excluding the C-terminal bundle domain. Hence, the structure of the Pex5p(C)-AGT complex supports the conformational changes of the receptor that have been observed previously on cargo binding [18],[19].

### Molecular Basis of AGT Recognition by the Pex5p Receptor

**Figure 2:**
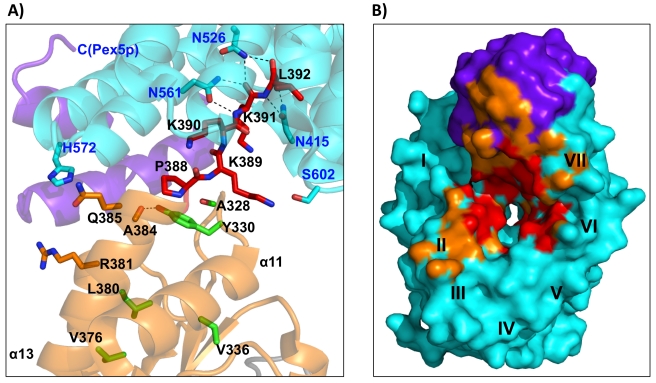
Structural basis of AGT recognition by the Pex5p receptor. (A) Zoom into the AGT-Pex5p(C) interface, depicting the side chains of several key residues from this interface. AGT residues labels are in black, and Pex5p residues labels are in blue. Additional AGT residues near the Pex5p interface, which are involved in hydrophobic core formation of the AGT C-terminal domain, are shown in green. Hydrogen bonds that are structurally conserved in both independent AGT-Pex5p complexes are shown with dashed lines. (B) AGT binding surface of the Pex5p receptor, which is almost 600 Å^2^ in size (cf., Table 3) and consists of a AGT PTS1 interface (red), surrounded by additional interface patches with other parts from AGT (orange). The approximate locations of the seven TPR repeats in Pex5p are labeled I–VII. There are additional non-PTS1 binding sites around TPR repeat II and the C-terminal bundle of the Pex5p receptor. The Pex5p complex that involves AGT chain A (cf., Figure 4) has been used for illustration. Color codes in both panels are as in Figure 1.

The structure of the AGT–Pex5p complex reveals that the C-terminal AGT domain (residues 283–392) is the exclusive and direct binding module of the Pex5p receptor (Figures 1 and 2). This domain comprises a bundle of the three helices α11 (residues 284–305), α12 (residues 332–343), and α13 (residues 370–387), in which the two longest helices (α11, α13) are in a parallel orientation to each other and the third (α12) crosses helix α13. The three helices are connected by a small two-stranded β-sheet (β8, residues 321–325; β9, residues 358–362) that forms an interface with the N-terminal catalytic AGT transaminase domain. The C-terminal sequence Pro-Lys-Lys-Lys-Leu (residues 388–392), corresponding to the PTS1, immediately follows helix α13.

The overall AGT–Pex5p interface consists of three distinct surface patches (Figures 1 and 2): the first involves the AGT-PTS1 (residues 389–392) that binds, as expected, into the central tunnel-like cavity of the ring-forming array of seven TPR segments of Pex5p(C), generating an interface of 550–600 Å^2^ (Interfaces Ia and Ib in Figure 1; Figure S5, left panel). The second includes the C-terminal part of the AGT helix α13 that immediately precedes the PTS1 (residues 381–388) and the loop connecting β9-α12 (residues 327–330) that interacts with this part of α13 (Interfaces IIa and IIb in Figure 1; Figure S5, central panel). We refer to this site in AGT as the “extended PTS1” interface, as it is directly upstream of the PTS1. Pex5p interactions from this interface overlap with hydrophobic core contacts by residues from α13 with other parts of the C-terminal AGT domain. Ala383, the most C-terminal AGT residue that is entirely buried within the AGT fold, is preceded by Arg381, which marks the most proximal residue in α13 that contributes to the extended PTS1–Pex5p interface. The third interface is topologically separate from the PTS1 and involves the loop that connects AGT helix α11 and strand β8 (residues 303–307) (Interfaces IIIa and IIIb in Figure 1; Figure S5, right panel). These two additional surface patches, when combined with the PTS1 binding site, increase the overall AGT–Pex5p(C) interface area by almost 2-fold, to around 1,000 Å^2^ (Table 3). A detailed structural description of all the three interface patches is provided in Text S1.

**Table 3:** Protein cargo recognition by the Pex5p receptor.

	AGT	SCP2
Molecular weight	43 kDa	15 kDa
Association state	dimer	monomer
Interface (Å^2^)	1,067, 966	1,005
PTS1 interactions	−11 (Arg381) to 0 (Leu392)	−7 (Leu136) to 0 (Leu143)
C-terminal hydrophobic core interaction	Cys387	Leu136
K_D_ (protein cargo) (µM)	3.5	0.11^a^
K_D_ (PTS1) (µM)	13.5^b^	0.66
K_D_ gain cargo protein versus peptide	3.9	6.0
Cargo fold upon Pex5p binding	Non-autonomous	Autonomous

a Data taken from [15].

b Data taken from [26].

The three binding sites are topologically preserved in the two AGT–Pex5p(C) modules. However, direct comparison reveals that when using the structure of Pex5p(C) as the basis of superposition, the orientation of the two bound AGT molecules deviate substantially (Figures S5 and S6). If the two protein components are assumed to be rigid bodies, the tilt and twist angles defining their relative orientation [20] change by 27 and 11 degrees, respectively. The difference originates from a limited conformational flexibility with a pivot point at the C-terminus of the AGT helix α13, preceding the PTS1 motif. Owing to the rigidity of the remaining AGT structure, the spatial differences in the superimposed complexes increase to around 20 Å in those parts of each AGT protomer that are most distal to the Pex5p(C) receptor-binding site (Figure S6). Because of these conformational differences, there is little conservation in the specific AGT–Pex5p(C) interactions. With the exception of a few conserved hydrogen bonds formed between three asparagines of Pex5p (Asn415, Asn526, Asn561) and the C-terminal main-chain carboxylate group of Leu392, along with the preceding peptide bond connecting Lys391 and Leu392, the remaining side chains of the AGT PTS1 sequence Lys389-Lys390-Lys391 are either not involved in further specific interactions or, if observed, these interactions are not conserved within the complete Pex5p-(AGT)_2_-Pex5p complex (Figure 2 and Figure S5). These findings are in agreement with an overall endothermic assembly process under the experimental in vitro conditions, indicating that AGT–Pex5p(C) complex formation is an entropy-driven process (Tables 1 and S1) rather than being dominated by specific enthalpic interactions.

### Conformational Changes in the AGT C-Terminal Domain Affect Pex5p Receptor Recognition

A key finding from our structural data is that binding of the AGT PTS1 motif to the Pex5p receptor is not autonomous from the additional cargo–receptor binding sites, both in terms of sequence connectivity and surface topology. These data could explain why many pathological AGT disease mutations that lead to AGT mistargeting are remote from the Pex5p-binding site. On the basis of our structural data, we argue that even minor folding defects or conformational alterations in AGT could compromise the binding of the AGT composite Pex5p interface, formed by the AGT C-terminal domain and PTS1.

To address this assumption, we mutated several residues in the AGT C-terminal domain close to the Pex5p-binding interface, which we expected to lead to conformational changes in this domain without compromising AGT activity (Figures 2 and S1). The first set of mutations involved two residues from the β9–α12 loop (Ala328, Tyr330) that interact with residues from the C-terminal helix α13 (Leu384 and Lys389). We introduced either more bulky side chains (A328W, Y330W) or removed side chain-specific intramolecular interactions (Y330A). For the second set of AGT variants, we aimed to affect the hydrophobic interactions of the C-terminal helix α13 with other parts of the AGT C-terminal domain. For this purpose, we mutated two residues from this helix (Val376, Leu380) that are completely buried into either an aspartate or proline. Additionally, to provide a structural rationale for established AGT disease mutations, we selected two AGT single residue polymorphisms (G170R, V336D) and the corresponding AGT double mutant G170R/V336D, which have been found in combination with the minor allele haplotype (*AGXT-Mi*) in PH1 patients. The AGT double mutant G170R/V336D results in a serious pathogenic effect and is non-responsive to pyridoxine treatment [2],[4]. However, the disease-causing mechanism of this AGT polymorphism, like various other mutations, has remained enigmatic. More specifically, the aggravating effect of the V336D mutation from the C-terminal domain in conjunction with the widespread G170R mutation seemed to be inexplicable, as the latter (G170R) is coupled with unwanted mitochondrial import in the *AGXT-Mi* isoform [21], again by an unknown mechanism of action. A structure of the AGT G170R mutant revealed only minor local conformational changes [22].

First, we attempted to purify all the AGT mutants to test their ability to bind the Pex5p receptor in vitro and to measure their catalytic activities (Table 1). However, the AGT variants with mutations in residues of the C-terminal helix α13 (Val376, Leu380) were insoluble when overexpressed in *Escherichia coli*, demonstrating that the hydrophobic core interactions of helix α13 are essential for proper folding of the enzyme under the chosen experimental conditions. The same problem of aggregation arose for the pathogenic AGT double mutant G170R/V336D, whereas each of the two single residue variants (G170R, V336D) could be expressed in significant quantities as soluble proteins. Although the aggregated AGT mutants could not be further characterized in vitro, they were used in functional assays to assess their tendency for aggregation in vivo and to investigate the level of peroxisomal targeting from AGT versions with suspected folding defects (see below).

All remaining mutants were purified by affinity chromatography and gel filtration (Figure S3). Proper folding of each protein was confirmed by far-UV circular dichroism spectroscopy (Figure S3). These AGT mutants had catalytic activities similar to the wild-type enzyme irrespective of Pex5p binding with the exception of the G170R mutant, which showed a decrease in activity of around 25%, in qualitative agreement with previous data [17]. Whereas the two pathogenic AGT single-residue mutants (G170R, V336D) did not show a significant change in Pex5p receptor binding, the AGT variants with mutations in the β9–α12 loop showed 2- to 6-fold decreased binding affinities for the Pex5p receptor when compared with the wild-type enzyme (Table 1). The weakest interaction, with a K_d_ of 19.4±8.3, was found for the Y330A AGT variant, indicating an important contribution of the side chain of Tyr330 to keep the β9-α12 loop in a conformation that is competent for Pex5p receptor binding. As for wild-type AGT, Pex5p binding by all of the AGT mutants is endothermic under the in vitro experimental conditions (Table S1).

**Figure 3:**
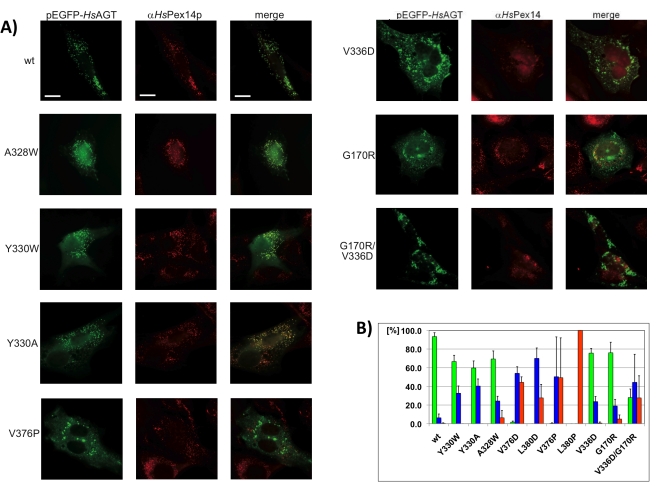
Subcellular distribution of AGT variants. (A) Human fibroblast cells were transfected with plasmids expressing EGFP-fused AGT variants, which are labeled. Congruent punctuate fluorescence pattern of wt-EGFP-AGT and Pex14p demonstrates peroxisomal localization. Additional diffuse cytosolic staining obtained for all mutants indicates partial cytosolic mislocalization. Large fluorescent patches that are not congruent with the characteristic peroxisomal pattern are the result of protein aggregation. Size bars, 10 µm. Additional images are shown in Figure S7. (B) Histogram showing the percentages of transfected cells displaying distinct EGFP-AGT localization patterns (green, peroxisomal; blue, cytosolic; red, aggregates). Standard deviations are shown with error bars.

To test the functional properties of all selected AGT variants in vivo, we employed a protein import assay in human fibroblasts, using enhanced green fluorescent protein (EGFP)-tagged AGT. When expressing EGFP-AGT without further modification in fibroblasts, we observed that more than 90% of the cells exhibited a punctuated pattern of peroxisomal localization (Figure 3). By contrast, a control version of the enzyme without the PTS1 (ΔPTS1) was evenly distributed in the cytosol without any visible sign of peroxisomal import (Figure S7), confirming that the presence of a PTS1 in AGT is crucial for recognition by the Pex5p receptor.

None of the AGT helix α13 variants showed significant measurable peroxisomal translocation, suggesting that fold defects in AGT lead to an almost complete loss of Pex5p import (Table 1; Figure 3). Aggregation of these AGT versions under in vivo experimental conditions is reflected by the formation of large fluorescent plaques in the cytosol, which are abundant in 28%–100% of transfected cells. This indicates substantial variability depending on the AGT mutant investigated. Whereas AGT(L380P), for instance, aggregates completely (Figure 3B), other AGT mutants (V376D, L380D) reveal a predominantly cytosolic background, suggesting a soluble cellular state with no peroxisomal association (Figure S7). These observations indicate that both misfolding and local conformational changes in the C-terminal domain have a synergistic effect, leading to a loss of AGT targeting to peroxisomes. The data also suggest that indirect effects, arising from altered structural properties of the AGT cargo, rather than direct and specific receptor interactions, are sufficient to abolish proper cargo recognition by the Pex5p receptor for peroxisomal targeting.

A slightly milder effect was observed with the pathogenic double mutant G170R/V336D, with 28% of the protein-forming plaques in the cytosol, and another 28% being properly translocated into peroxisomes (Figure 3). The overall level of non-peroxisomal localization of this AGT mutant is 72%. All remaining AGT variants, including those from the β9–α12 loop and the two pathogenic single-residue mutants (G170R, V336D)< displayed 59%–76% peroxisomal localization, which is in agreement with our in vitro binding data and indicates a weakening but not an abolishment of Pex5p binding. Two AGT mutants from this category (G170R, A328W) showed around 5% aggregation, whereas no significant level of aggregation was measured for the remaining mutants. Taken together, the data show that even minor structural perturbations in AGT have a measurable and significant effect on AGT translocation.

## Discussion

### AGT Is Recognized by the Pex5p Receptor as Oligomeric and Functional Cargo

The AGT–Pex5p structure is the second cargo protein–Pex5p receptor complex determined to date, the first being sterol carrier protein 2 (SCP2)–Pex5p [15]. Our data indicate that the dimeric and cofactor-bound arrangement of AGT is preserved and that the enzyme remains functional prior to and upon binding of the Pex5p receptor (Table 1). This observation is in agreement with the unique ability of peroxisomes to import even large and oligomeric cargos as functional protein assemblies [23]–[25]. As our studies have been carried out in the absence of any additional protein components, a potential requirement of adaptor proteins as previously suggested [9],[11] is unlikely. Our data confirm the involvement of segments from the C-terminal AGT domain—previously described as the “PTS1A” binding site [9]—in Pex5p receptor binding, but do not support earlier suggestions that an N-terminal AGT sequence segment contributes to receptor recognition [10].

### Non-Autonomous PTS1 Recognition by the Pex5p Receptor

Comparison of the complexes of Pex5p with SCP2 and AGT allows for the first time the identification of common and diverging principles in target protein recognition (Table 3), beyond the well-established C-terminal PTS1 motif that is shared by most Pex5p cargos [26]. Notably, the measured AGT–Pex5p interaction is about 30-fold weaker than that observed for SCP2. This argues in favor of AGT being highly sensitive to perturbations that affect Pex5p recognition (Table 1; Figure 3) and may mirror the large number of known disease-causing AGT mutations that have been associated with protein mistargeting rather than with catalytic activity effects [4].

**Figure 4:**
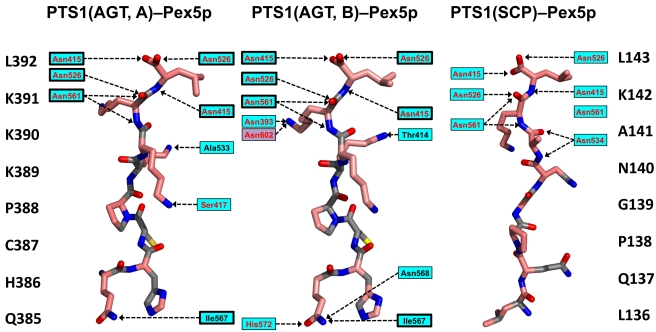
Comparison of PTS1–receptor interactions, based on the receptor complexes with AGT (chains A and B) and SCP2. The respective PTS1 sequences are shown to the left and to the right. Hydrogen bonds with the receptor are indicated with dashed lines (black characters, main-chain interactions; red characters, side-chain interactions; background colors are as in Figure 1). Those interactions in the AGT–Pex5p complex that are structurally conserved are boxed.

The two protein cargo–receptor complex structures reveal that there are almost no specific, conserved side-chain interactions between polar residues from each PTS1 motif with Pex5p, with the notable exception of the very C-terminal leucine residue (Figure 4). This observation is supported by previous findings on AGT that indicate side-chain tolerance at PTS1 position −3 and, albeit more limited, at position −1 [27]. By contrast, our data only partly agree with observations from Pex5p–PTS1 peptide complexes, in which a more extensive hydrogen bond network over several PTS1 residues was observed [25],[28],[29]. Comparison with the available Pex5p–cargo protein complex structures indicates that the adaptability of possible PTS1 conformations to optimize specific interactions with the Pex5p receptor is restricted owing to the additional non-PTS1 protein interfaces that are formed between the C-terminal bundle domain of the receptor and cargo, as previously shown for SCP2 [15],[30] and for AGT in this contribution (Figure 2). Collectively, however, the additional non-PTS1 interactions (marked as IIa,b and IIIa,b in Figure 1) only slightly enlarge the overall Pex5p–AGT interface, in comparison to that observed in the Pex5p-SCP2 complex, in one of the two Pex5p-AGT complexes (Table 3).

The specific Pex5p binding abilities of the PTS1 cargo peptides, corresponding to AGT and SCP2 sequences, are weak, with dissociation constants in the low to sub-µM range [15],[26]. The gain in binding affinity for AGT when the complete protein is used is around 4-fold—3.5 µM instead of 13.5 µM (Table 3). Similarly, a gain in binding affinity of around 6-fold has been found for SCP2–Pex5p assembly when the protein complex is compared with the corresponding PTS1 peptide complex [15]. However, a recent analysis of additional non-PTS1 interactions confirmed that their contribution is only of minor importance, in turn suggesting that SCP2 recognition by the Pex5p receptor is principally driven by autonomous recognition of its PTS1 motif [30]. By contrast, our structural and functional data on AGT–Pex5p show that complex formation is both dependent on the presence of the AGT PTS1 motif and the correct Pex5p binding-competent conformation of the AGT C-terminal domain. Based on these findings, we argue that previously reported problems in establishing in vitro binding with purified protein components and by transfection experiments have failed for several PTS1 protein cargos in vivo owing to contextual defects in protein folding and possibly oligomerization [31],[32]. Moderate binding of the cargo in vivo may facilitate subsequent release of the cargo into the peroxisomal lumen, a process that at present is still less well understood than the mechanism of cargo binding [18],[33],[34].

**Figure 5:**
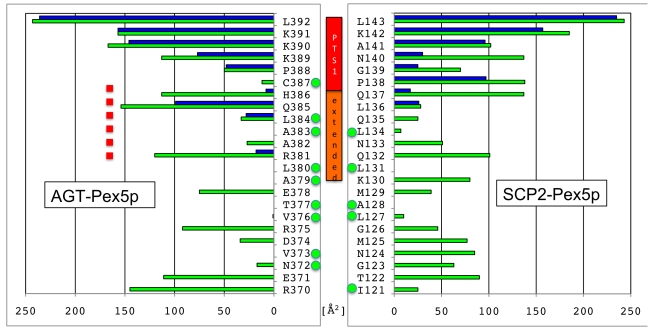
Comparison of Pex5p receptor and hydrophobic core interactions in SCP2 (left) and AGT (right). Green bars, accessible surface area per residue; blue bars, interaction surface with Pex5p receptor. Residues with side chains involved in cargo hydrophobic core are labeled with green dots. In contrast to SCP2, there is a seven-residue segment in AGT (381–387) that contributes to the core of the AGT fold and to Pex5p receptor interactions (red dashed line).

Further investigation of our structural data of the AGT–Pex5p complex reveals that the sequence segments in AGT that constitute the PTS1 and the hydrophobic core of AGT topologically overlap, whereas in SCP2 the corresponding sequence segments are well separated (Figure 5). Specifically, the PTS1 interactions observed extend to Arg381 (PTS1 position −11, when considering the C-terminal Leu392 as position 0), and the side chains of three residues within the extended PTS1 segment (Ala383, Leu384, Cys387) are also involved in hydrophobic core interactions of the C-terminal AGT domain. The overlapping interactions thus generate a seven-residue segment (381–387) from the C-terminus of helix α13 (Figure 2A) [8] that is involved in both the overall AGT fold and Pex5p receptor recognition. These structural observations indicate that, in contrast to our previous findings on the SCP2–Pex5p complex [15], Pex5p receptor recognition of the PTS1 in AGT is structurally non-autonomous with respect to the remaining fold of the enzyme. Our structural data also explain previous observations on the translocation of AGT molecules that contain mutations in the extended PTS1 motif. These studies showed that diminished binding is caused by folding defects rather than by loss of cargo–receptor interactions that were predicted prior to available structural data [27], indicating that AGT PTS1 binding depends on properly folded AGT and thus is also functionally non-autonomous. This is well illustrated by the strong translocation defects of several extended PTS1 mutations in AGT (L380P; V376P) [27], which are involved in AGT hydrophobic core interactions rather than specific AGT–Pex5p interactions (Figure 5).

In AGT, the additional Pex5p non-PTS1 interactions observed are not as specific as one may expect (Figures 2A and S5) and perhaps explain the moderate overall binding affinity. These findings are further supported by the observation that Pex5p–AGT binding in vitro is an entropy-driven process, suggesting that binding is dominated by order/disorder processes rather than by enthalpy-driven specific interactions.

### Towards New Approaches to Restore AGT Function in Disease Treatment

AGT is an enzyme with a well-established genotype/phenotype database, including about 150 different missense mutations, many of which lead to serious forms of PH. Our structural and functional characterization of the molecular parameters for AGT to be recognized by the Pex5p receptor and its subsequent translocation into peroxisomes offers an opportunity to rationally address functional implications of pathogenic PH-causing missense mutations.

We assume that those PH mutations that lead to irreversible AGT aggregation, irrespective of the presence of the Pex5p receptor, will be difficult or even impossible to treat by chemical intervention as these AGT variants are expected to lose both their enzymatic activity and their ability to be recognized by the peroxisomal Pex5p receptor as a consequence of misfolding. Based on our mapping of known AGT missense mutations on the three-dimensional structure of the AGT–Pex5p complex (Figure S1), we estimate that around half of these lead to fold defects, as they reside in regions that are completely buried within the AGT fold. The fraction of misfolded AGT mutants is probably even higher when associated with the widespread *AGXT-Mi* gene [3], which leads to additional destabilization of the enzyme. Partial rescue of some of these mutations, by adding chaperones or osmolytes for instance [35], may be possible but remains challenging, as most of these additives tend to be non-specific.

On the basis of our data, we further expect that the loss of function of many of the remaining patient mutations (Figure S1) that result in AGT, which does not aggregate or is only partially prone to aggregation, could be potentially restored by proper chemical intervention. As the topology of the AGT active site is well characterized by PLP binding (Figure S4) and the presence of several highly conserved residues, mutations that directly affect AGT enzymatic activity are predictable and their effect can be verified by AGT activity tests [4],[5]. For mutants of this category, it has been shown that pyridoxine treatment may lead to additional active-site stabilization, resulting in a reduction of clinical symptoms such as calcium oxalate crystallization and an increasing preservation of renal function [36].

However, prior to this work, a rational basis for predicting mutations involved in the loss of peroxisomal targeting has been largely missing. A paradigm pathogenic mutation within this category is the G170R/V336D variant located on the *AGXT-Mi* allele [36], which creates a serious disease phenotype. For this type of mutation, which predominantly affects peroxisomal targeting, it is desirable to identify compounds that would lead to a gain in AGT binding to the Pex5p receptor, by targeting identified AGT–Pex5p interface areas such as the PTS1 site, the extended PTS1 site, and relevant Pex5p-binding surfaces from the AGT C-terminal domain (Figures 1–2 and S5). The knowledge of designed AGT variants compromised in Pex5p recognition, such as AGT(Y330A), may be useful for targeting the restoration of AGT–Pex5p recognition to wild-type levels. The observed limited flexibility in the non-PTS1 binding areas and the lack of optimized interactions within the PTS1 binding site of AGT (Figures S5 and S6) may provide a knowledge-based system by which Pex5p receptor binding can be maximized by compounds that have the potential to improve protein-protein interactions.

## Materials and Methods

### Cloning and Protein Expression

Human AGT (major allele haplotype) and human Pex5p(C) (residues 315–639) were expressed from a modified pET24d vector (G. Stier, EMBL Heidelberg) in *Escherichia coli* BL21(DE3) RIL. The two genes were amplified by polymerase chain reaction (PCR) using primers containing NcoI and KpnI restriction sites, respectively (Table S4). Following the digestion of the PCR products and the vector, the two constructs were created by ligation (Rapid Ligation Kit, Fermentas). Cultures were grown in Lysogeny Broth medium containing 50 mM Tris pH 7.5 and 1% (w/v) glucose, and induced mid-log phase with 0.5 mM isopropyl-β-D-thiogalactopyranosid overnight at 21°C. Both proteins contained an N-terminal hexahistidine–glutathione S-transferase fusion, which is cleavable with tobacco etch virus (TEV) protease. The cleared lysate was loaded onto a nickel-nitrilotriacetic acid column and the purified proteins were eluted with 50 mM Tris pH 8.0, 150 mM NaCl, 2 mM ß-mercaptoethanol, and 500 mM imidazole. Fusion proteins were cleaved with tobacco etch virus protease overnight at 4°C, along with dialysis into 50 mM Tris pH 8.0, 150 mM NaCl, 2 mM ß-mercaptoethanol, and 20 mM imidazole. The samples were then applied to a nickel-nitrilotriacetic acid column and the flow-through was collected. As a final purification step, gel filtration was performed using a Superdex 75 (16/60) column (GE Healthcare).

In vivo analysis of EGFP-AGT was carried out with the expression vector pEGFP-AGT, which was derived from subcloning a PCR amplification product of AGT into the pEGFP-C1 plasmid (Clontech). Point mutations were introduced into pEGFP-AGT by using the Quickchange XL Site Directed Mutagenesis Kit (Stratagene). All primers are listed in Table S3.

AGT point mutants that were tested in vitro were subcloned into a pET151 D-TOPO vector. Expression and purification of these proteins was performed as described above.

### Complex Formation, Characterization, and Enzyme Assay

The Pex5p(C)–AGT complex was formed by mixing purified Pex5p(C) and AGT and confirmed by analytical gel filtration and static light scattering, using a MiniDAWN instrument (Wyatt). Specific activity measurements of AGT in the presence and absence of Pex5p were performed as described previously [37],[38], using the following concentrations: 100 mM potassium phosphate pH 8.0, 0.15 mM PLP, 10 mM glyoxylate, and 150 mM alanine. To confirm specific binding of the cofactor to the recombinant enzyme, we recorded absorption spectra between 300 and 600 nm. All measurements were performed on an Infinity 1000 spectrophotometer (Tecan).

### Crystallization and X-Ray Structure Determination

Pex5p(C) and AGT were mixed in a 3∶2 molar ratio and concentrated to 5 mg/ml. Crystals were obtained by submitting a mix of 1 µl protein and 1 µl reservoir solution, comprising 0.1 M Bis-Tris (pH 5.3), 0.15 M LiSO_4_, 17% [w/w] PEG3350, to hanging drop vapor diffusion at 20°C. Streak seeding of a drop with 2.5 mg/ml protein concentration was used to obtain single large crystals.

X-ray data were collected at BM14.1 at ESRF, Grenoble. Data were processed with MOSFLM [39] and scaled with SCALA [40]. Five percent of the reflections were randomly selected for cross-validation. The structure of the Pex5p(C)–AGT complex was solved by molecular replacement using the coordinates of apo-AGT (PDB code: 1H0C) and the Pex5p–SCP2 complex (PDB code: 2C0L) as search models with the program PHASER [41]. REFMAC [42] was used to refine the structure, applying translation/libration/screw parameterization [43]. Manual building and structure analysis were carried out in COOT [44]. The structure quality was assessed with MOLPROBITY [45]. Programs of the CCP4 package [46] were used for structure manipulation, analysis, and validation. The coordinates of the structure have been deposited in the Protein Data Bank (code: 3R9A). Tilt and twist angles were calculated using MOD22 [20].

### Isothermal Titration Microcalorimetry

All proteins were dialyzed against 100 mM HEPES (pH 7.5), 150 mM NaCl, and 2 mM ß-mercaptoethanol. ITC measurements were conducted on a MicroCal VP-ITC using 25–46 µM AGT as a sample and 250–460 µM Pex5p(C) as a titration ligand. Experiments were performed at 25°C. Pex5p(C) was injected in volumes of 10 µl in a total of 27 steps, resulting in a 2-fold excess of AGT at the end of each titration experiment. Ligand heating effects by dilution were subtracted, and data were fitted using MicroCal Origin 5.0.

### Circular Dichroism

Circular dichroism experiments were performed on a J-810 spectropolarimeter (Jasco). Proteins were dialyzed into 10 mM potassium phosphate (pH 8.0) and 1 mM dithiothreitol. Far-UV spectra were recorded between 190 and 260 nm, using a 1 mm cuvette and a concentration of 0.15–0.22 mg/ml protein, as determined by specific absorbance at 280 nm. The machine settings were 1 nm bandwidth, 1 s response, 1 nm data pitch, and 100 nm/min scan speed. Secondary structure content was calculated with the Diochroweb server [47], using the analysis program CDSSTR and reference set 4. All circular dichroism data presented are the averages of three separate experiments.

### Localization Assay

Human fibroblast cells (strain GM5756T) were cultured as described previously [15] and transfected with pEGFP-AGT variants, using FuGENE 6 Transfection Reagent (Roche Diagnostics). At 24 h after transfection, cells were fixed with 3% paraformaldehyde, solubilized with 1% Triton X-100, and subjected to immunofluorescence microscopy. Polyclonal rabbit antibodies against Pex14p were used to label peroxisomes [48]. Secondary antibodies were conjugated with Alexa Fluor 594 (Invitrogen, Germany). All micrographs were recorded on an Axioplan 2 microscope (Zeiss) with a Plan-Apochromat 63×/1.4 oil objective and an Axiocam MR digital camera and were processed with AxioVision 4.6 software (Zeiss). Statistical analysis was carried out from at least three independent transfections of each AGT expression plasmid. Based on the appearance of the AGT fluorescence pattern, around 100 cells of each experiment were visually categorized into three classes: (i) predominant peroxisomal localization, (ii) mostly cytosolic, or (iii) forming aggregates, as indicated by fluorescent plaques over cytosolic background.

## Supporting Information

Figure S1 Missense mutations and designed mutations in AGT. In the green/orange AGT protomer, known missense mutations are shown by red spheres, indicating that they are distributed over the entire AGT structure. In the grey/orange AGT protomer, designed single residues and selected missense mutations are shown with cyan and blue spheres, respectively, and are labeled. The two PLP cofactors are indicated in stick presentation, using atom-specific colors. The color code of the shown ribbon is as in Figure 1.
(TIF)

Figure S2 Stereo image of the PTS1 of AGT (residues 385–392), within a final 2Fo-Fc electron density at 1σ contour level. The structural framework of the Pex5p(C)-AGT complex is indicated by a cartoon presentation in faint colors, using the color codes of Figure 1.
(TIF)

Figure S3 Biophysical characterization of AGT variants. (A) Purity of AGT mutants were analyzed by SDS-PAGE. Lane annotations: Marker; 1, AGT(wt); 2, AGT(A328W); 3, AGT(Y330A); 4, AGT(Y330W); 5, AGT(V336D); 6, AGT(G170R). (B) Analysis of the oligomeric state of the Pex5p(C)-AGT complex and single protein components by analytic gel filtration and static light scattering. Color codes: Pex5p(C), green; AGT, blue; Pex5p(C)-AGT, red. The molecular weights of the peaks in the elution profiles are measured at 34.2 kDa, 83.4 kDa, and 142 kDa, respectively, which correlate well with the calculated molecular weights of Pex5p(C) (36 kDa), AGT (86 kDa), and a 2∶2 Pex5p (C)∶AGT complex (158 kDa). (C) Circular dichroism spectra for purified AGT mutants (cf., panel A). The estimated secondary structural content for each mutant is summarized in Table S3.
(TIF)

Figure S4 Evidence for the presence of a Lys209-PLP adduct in the Pex5-AGT complex. (A and B) Lys209-PLP (yellow) interaction network in AGT chains A (green) and C (grey), respectively, within the AGT-Pex5p complex. Residues from the other AGT protomer within each of the two PLP-binding sites are shown in chain-specific colors. The final electron density map for each Lys209-PLP is shown at 1.0σ contour level. Interacting residues are shown in stick representation and are labeled. (C) Absorption spectra were recorded for AGT protein solutions at pH 7.5 (green) and pH 5.3 (red), which represents the pH used for crystallization. Maximum absorbance was measured at 420 nm, indicating the presence of an internal aldimine, as observed in the crystal structure (cf., panels A and B).
(TIF)

Figure S5 AGT-Pex5p interface patches, divided in three areas entitled “PTS1,” “extended PTS1,” and “C-terminal domain.” Both AGT-Pex5 interfaces from the overall Pex5p-(AGT)_2_-Pex5p complex are shown separately (upper and lower panel) and are labeled (Ia, Ib, IIa, IIb, IIIa, IIIb) according to Figure 1. Interacting residues are shown in stick presentation and are labeled. Hydrogen bonds are shown with dashed lines. Note that various interactions are not conserved in the two AGT-Pex5p complexes. Color codes are as in Figure 1.
(TIF)

Figure S6 Differences in the overall arrangement of the two AGT-Pex5 complexes. The two complexes are superimposed using the coordinates of the Pex5p receptor and are shown in two different orientations, differing by 90° around a vertical axis. Spatial differences between the two AGT molecules bound to Pex5p are indicated to the left and are translated into distance range-specific color codes in one of the two superimposed AGT molecules, whereas the other one is colored in grey. The difference of the Pex5p–AGT arrangement in the two complexes has also been measured by a tilt/twist angle analysis.
(TIF)

Figure S7 Localization of additional AGT variants. Human fibroblast cells were transfected with plasmids expressing EGFP-fused AGT variants. Truncation of the C-terminal PTS1 of AGT results in non-punctuated diffuse staining, indicating cytosolic mislocalization (ΔPTS1). The other AGT mutants (V376D, L380D, L380P) form large fluorescent patches that are not congruent with the characteristic peroxisomal pattern (αHsPex14) suggesting protein aggregation. Size bars, 10 µm.
(TIF)

Table S1 Quantitative determination of Pex5p interaction with AGT mutants by isothermal titration microcalorimetry. The average stoichiometry calculated from all performed measurements is 1.02±0.05.
(DOC)

Table S2 Comparison of the structure of AGT and Pex5p.
(DOC)

Table S3 Estimated secondary structure content of purified AGT variants.
(DOC)

Table S4 Primer sequences used.
(DOC)

Text S1 Detailed description of AGT-Pex5p interface interactions.
(DOC)

## Abbreviations

AGT: alanine-glyoxylate aminotransferase
*AGXT-Mi*: minor allele haplotype of *AGT*
EGFP: enhanced green fluorescent protein
ITC: isothermal titration microcalorimetry
PCR: polymerase chain reaction
PH1: Primary hyperoxaluria type 1
PLP: pyridoxal-5′-phosphate
PTS1: peroxisomal targeting signal 1
SCP2: sterol carrier protein 2
TPR: tetratricopeptide repeat
