# Supplementary figures and images for: Molecular Requirements for Peroxisomal Targeting of Alanine-Glyoxylate Aminotransferase as an Essential Determinant in Primary Hyperoxaluria Type 1
Source: PLoS Biol. 2012 Apr 17;10(4):e1001309. doi: 10.1371/journal.pbio.1001309 (PMC3328432; doi:10.1371/journal.pbio.1001309)

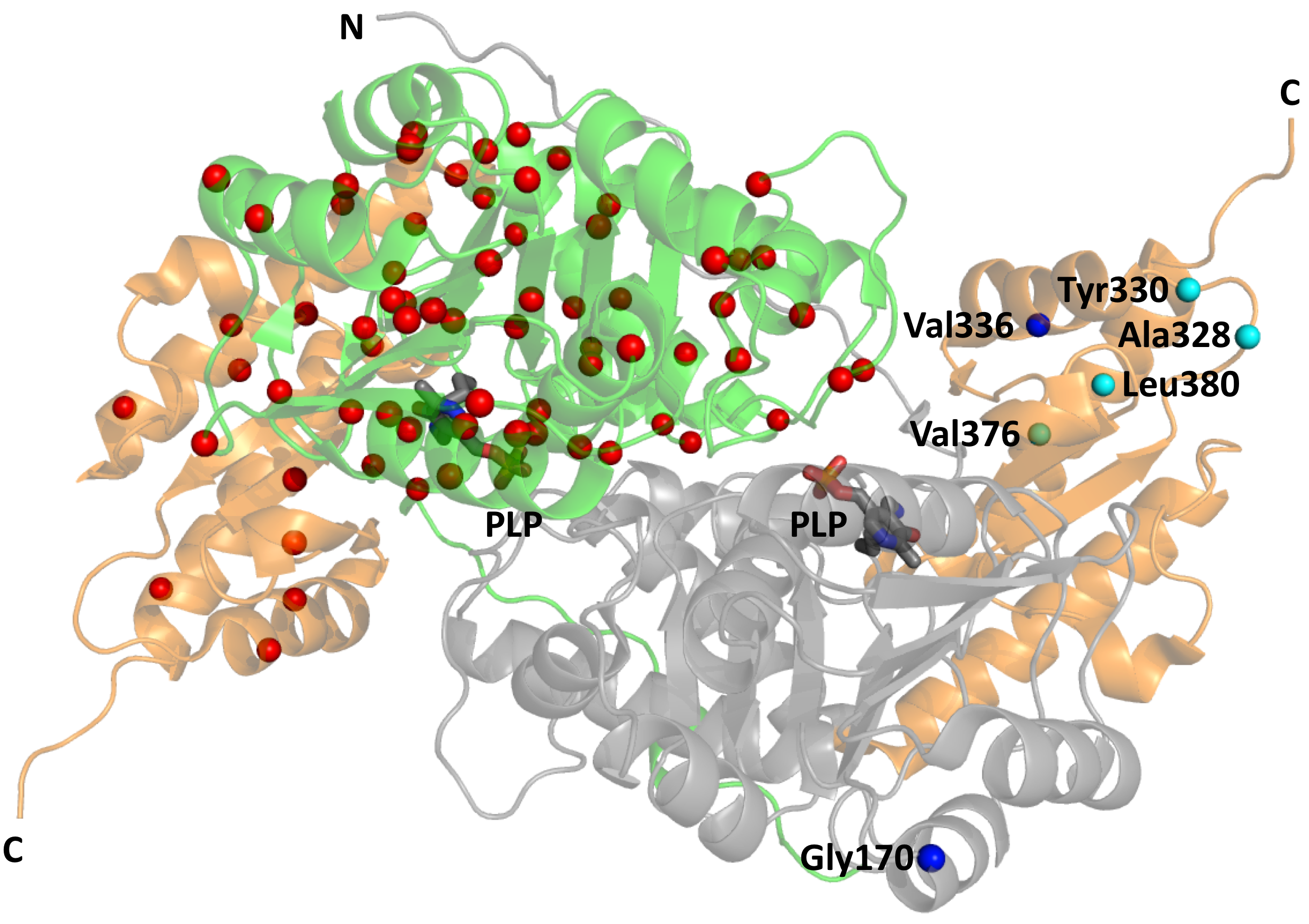

Supplement: Figure S1 — Missense mutations and designed mutations in AGT. In the green/orange AGT protomer, known missense mutations are shown by red spheres, indicating that they are distributed over the entire AGT structure. In the grey/orange AGT protomer, designed single residues and selected missense mutations are shown with cyan and blue spheres, respectively, and are labeled. The two PLP cofactors are indicated in stick presentation, using atom-specific colors. The color code of the shown ribbon is as in Figure 1. (TIF) [file pbio.1001309.s001.tif]

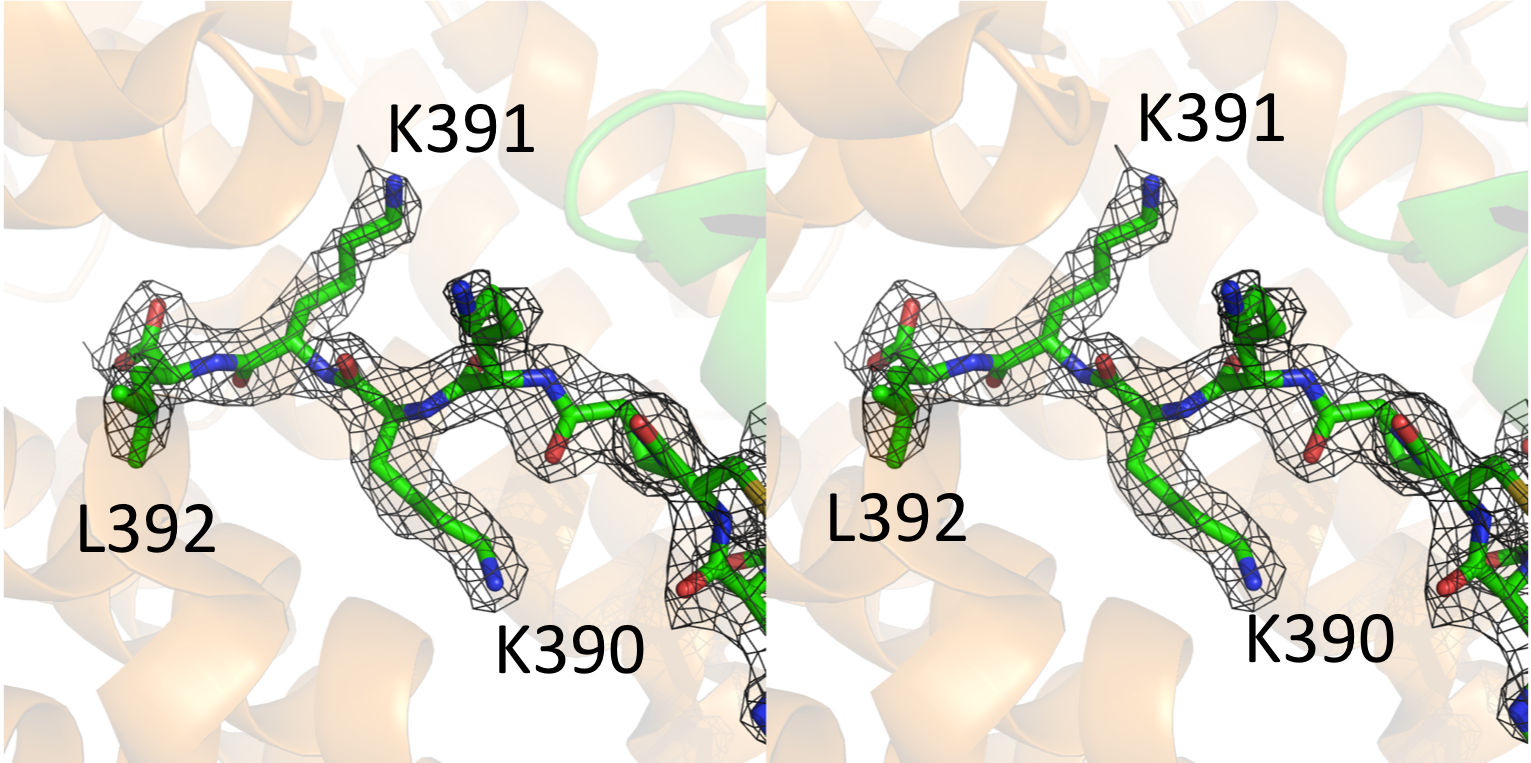

Supplement: Figure S2 — Stereo image of the PTS1 of AGT (residues 385–392), within a final 2Fo-Fc electron density at 1σ contour level. The structural framework of the Pex5p(C)-AGT complex is indicated by a cartoon presentation in faint colors, using the color codes of Figure 1. (TIF) [file pbio.1001309.s002.tif]

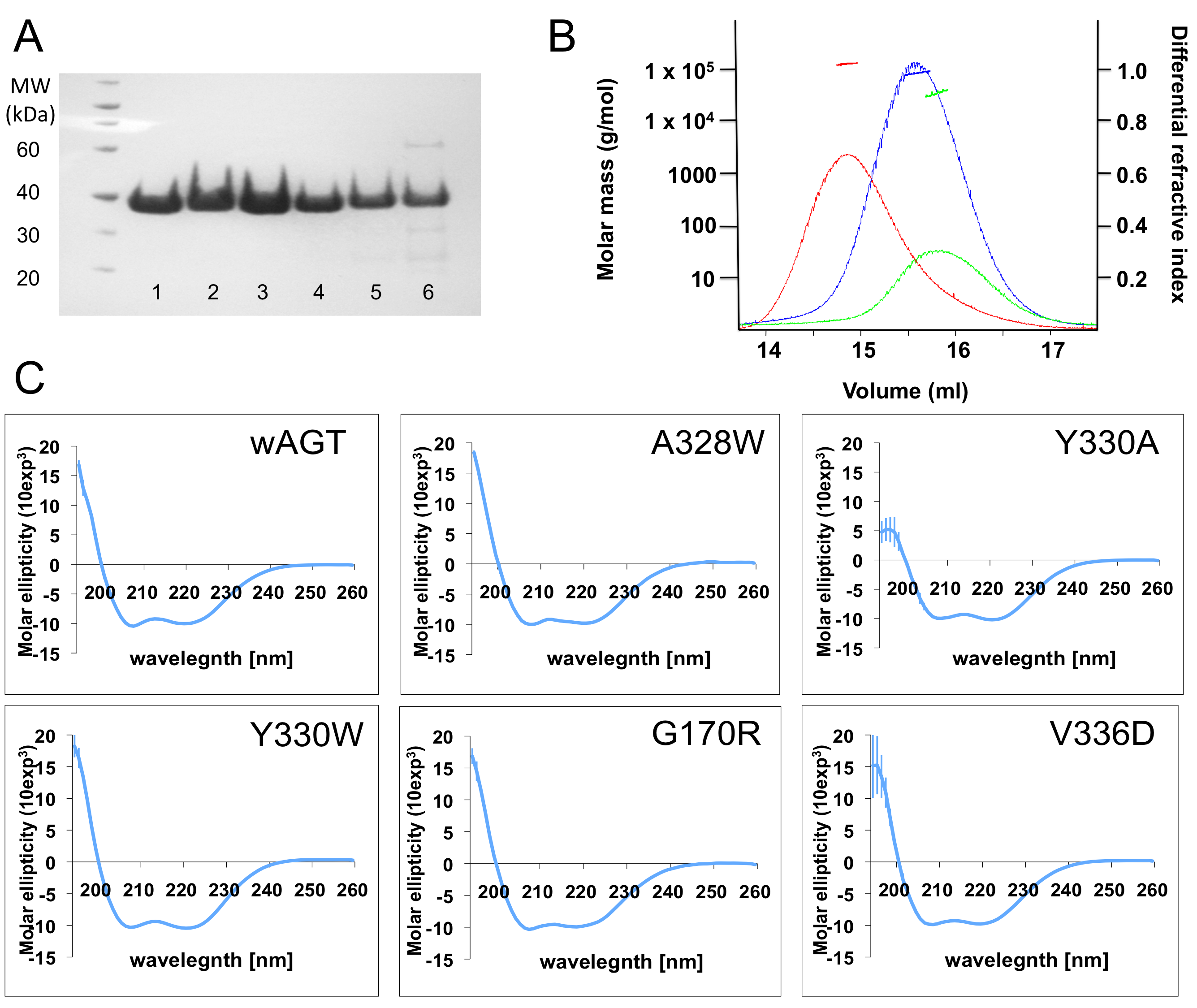

Supplement: Figure S3 — Biophysical characterization of AGT variants. (A) Purity of AGT mutants were analyzed by SDS-PAGE. Lane annotations: Marker; 1, AGT(wt); 2, AGT(A328W); 3, AGT(Y330A); 4, AGT(Y330W); 5, AGT(V336D); 6, AGT(G170R). (B) Analysis of the oligomeric state of the Pex5p(C)-AGT complex and single protein components by analytic gel filtration and static light scattering. Color codes: Pex5p(C), green; AGT, blue; Pex5p(C)-AGT, red. The molecular weights of the peaks in the elution profiles are measured at 34.2 kDa, 83.4 kDa, and 142 kDa, respectively, which correlate well with the calculated molecular weights of Pex5p(C) (36 kDa), AGT (86 kDa), and a 2∶2 Pex5p (C)∶AGT complex (158 kDa). (C) Circular dichroism spectra for purified AGT mutants (cf., panel A). The estimated secondary structural content for each mutant is summarized in Table S3. (TIF) [file pbio.1001309.s003.tif]

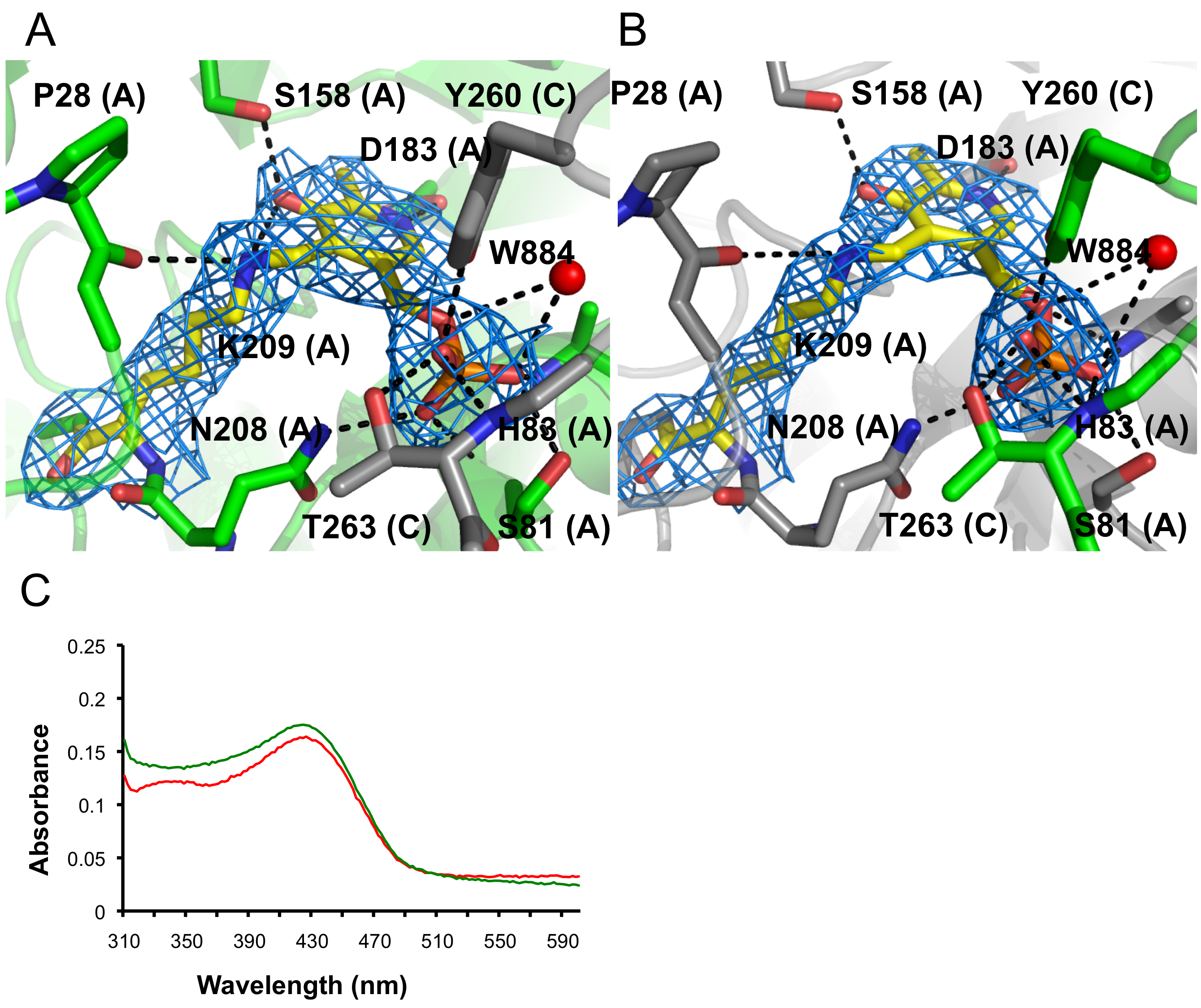

Supplement: Figure S4 — Evidence for the presence of a Lys209-PLP adduct in the Pex5-AGT complex. (A and B) Lys209-PLP (yellow) interaction network in AGT chains A (green) and C (grey), respectively, within the AGT-Pex5p complex. Residues from the other AGT protomer within each of the two PLP-binding sites are shown in chain-specific colors. The final electron density map for each Lys209-PLP is shown at 1.0σ contour level. Interacting residues are shown in stick representation and are labeled. (C) Absorption spectra were recorded for AGT protein solutions at pH 7.5 (green) and pH 5.3 (red), which represents the pH used for crystallization. Maximum absorbance was measured at 420 nm, indicating the presence of an internal aldimine, as observed in the crystal structure (cf., panels A and B). (TIF) [file pbio.1001309.s004.tif]

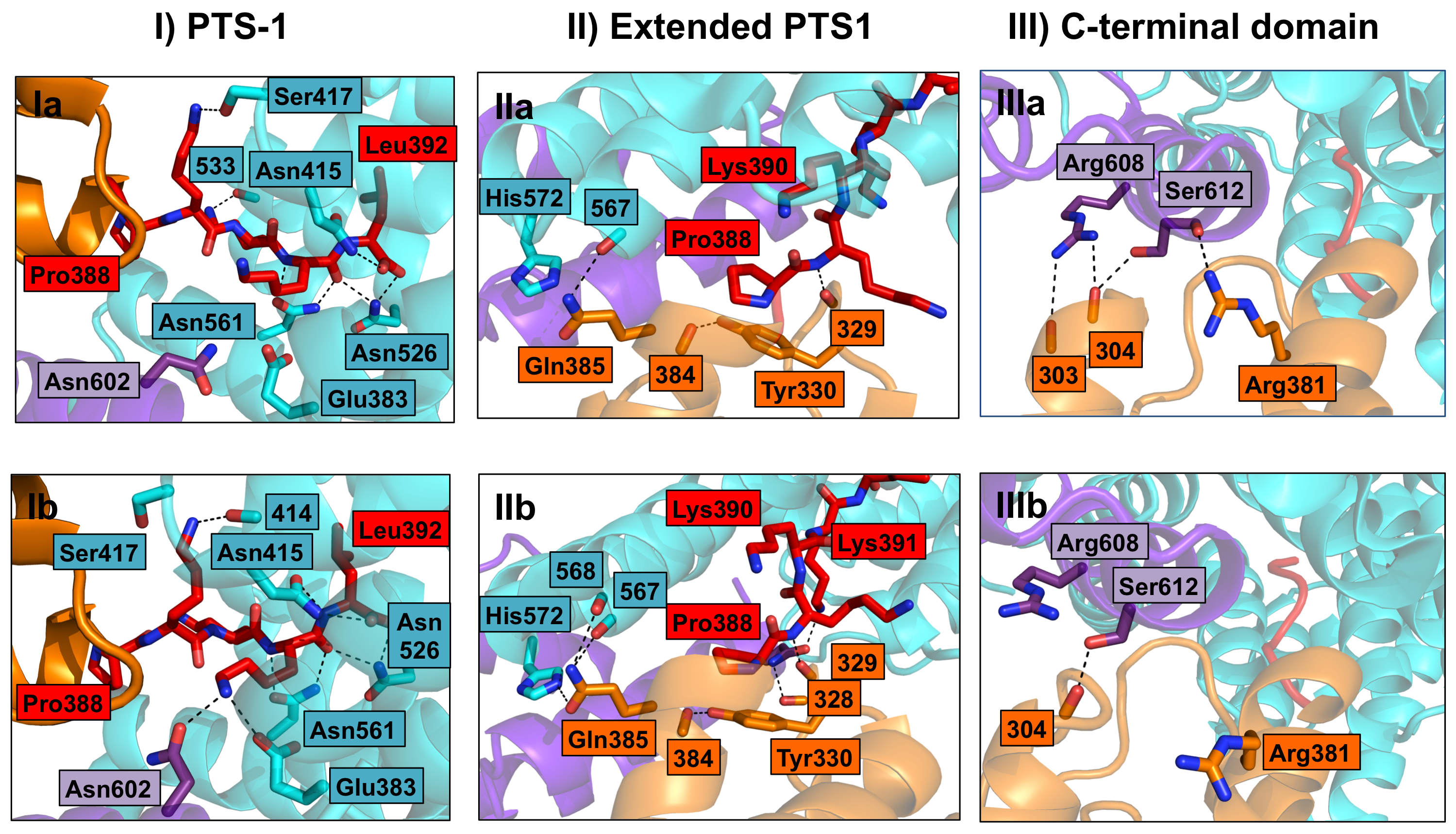

Supplement: Figure S5 — AGT-Pex5p interface patches, divided in three areas entitled “PTS1,” “extended PTS1,” and “C-terminal domain.” Both AGT-Pex5 interfaces from the overall Pex5p-(AGT)2-Pex5p complex are shown separately (upper and lower panel) and are labeled (Ia, Ib, IIa, IIb, IIIa, IIIb) according to Figure 1. Interacting residues are shown in stick presentation and are labeled. Hydrogen bonds are shown with dashed lines. Note that various interactions are not conserved in the two AGT-Pex5p complexes. Color codes are as in Figure 1. (TIF) [file pbio.1001309.s005.tif]

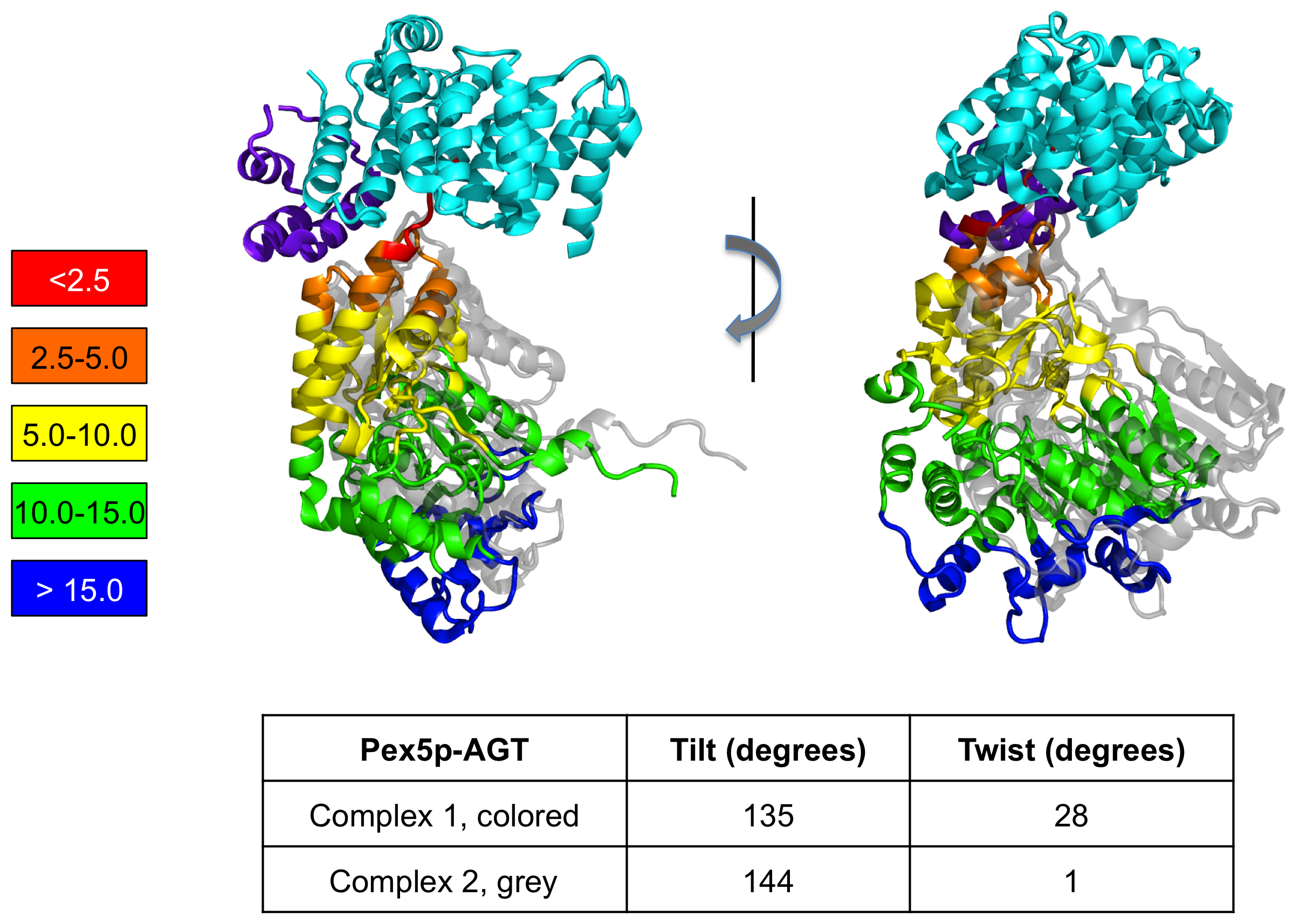

Supplement: Figure S6 — Differences in the overall arrangement of the two AGT-Pex5 complexes. The two complexes are superimposed using the coordinates of the Pex5p receptor and are shown in two different orientations, differing by 90° around a vertical axis. Spatial differences between the two AGT molecules bound to Pex5p are indicated to the left and are translated into distance range-specific color codes in one of the two superimposed AGT molecules, whereas the other one is colored in grey. The difference of the Pex5p–AGT arrangement in the two complexes has also been measured by a tilt/twist angle analysis. (TIF) [file pbio.1001309.s006.tif]

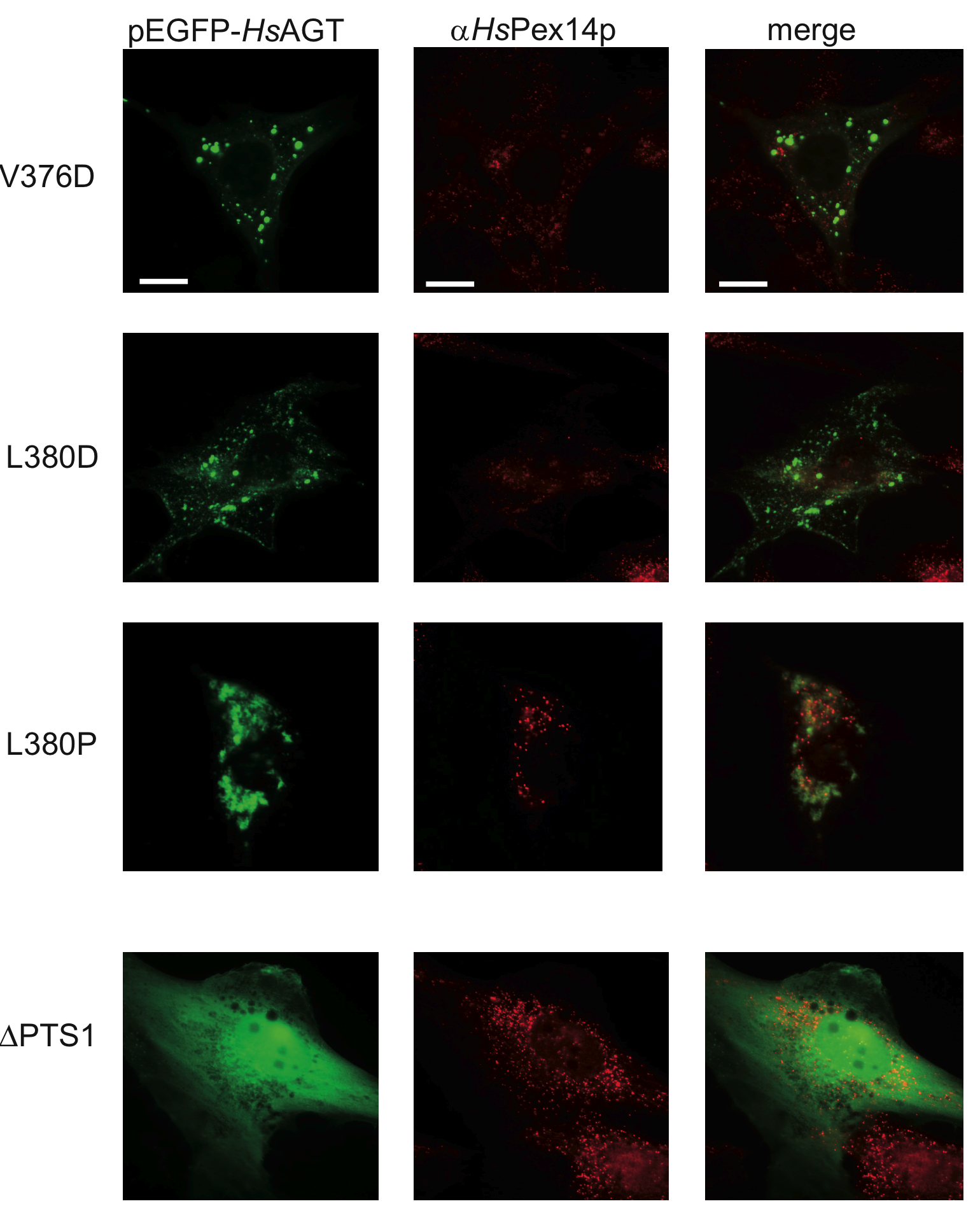

Supplement: Figure S7 — Localization of additional AGT variants. Human fibroblast cells were transfected with plasmids expressing EGFP-fused AGT variants. Truncation of the C-terminal PTS1 of AGT results in non-punctuated diffuse staining, indicating cytosolic mislocalization (ΔPTS1). The other AGT mutants (V376D, L380D, L380P) form large fluorescent patches that are not congruent with the characteristic peroxisomal pattern (αHsPex14) suggesting protein aggregation. Size bars, 10 µm. (TIF) [file pbio.1001309.s007.tif]
