# Supplementary material for: Molecular Requirements for Peroxisomal Targeting of Alanine-Glyoxylate Aminotransferase as an Essential Determinant in Primary Hyperoxaluria Type 1
Source: PLoS Biol. 2012 Apr 17;10(4):e1001309. doi: 10.1371/journal.pbio.1001309 (PMC3328432; doi:10.1371/journal.pbio.1001309)
Supplement: Table S1 — Quantitative determination of Pex5p interaction with AGT mutants by isothermal titration microcalorimetry. The average stoichiometry calculated from all performed measurements is 1.02±0.05. (DOC) [file pbio.1001309.s008.doc]

**Table S1:** Quantitative determination of Pex5p interaction with AGT mutants by isothermal titration microcalorimetry

| **Cargo** | **K_d_ (µM)** | **∆H (kJ/mol)** | **T∆S (kJ/mol)** | **∆G (kJ/mol)** |
| --- | --- | --- | --- | --- |
| **AGT(wt)** | 3.5 ± 0.4 | 15.9 ± 1.9 | 47.0 ± 2.0 | -31.1 ± 0.3 |
| **AGT(A328W)** | 6.2 ± 0.8 | 26.4 ± 4.9 | 56.1 ± 4.7 | -29.7 ± 0.3 |
| **AGT(Y330A)** | 19.4 ± 8.3 | 16.6 ± 3.7 | 43.6 ± 3.4 | -27.0 ± 0.1 |
| **AGT(Y330W)** | 7.9 ± 1.0 | 23.4 ± 2.5 | 52.6 ± 2.8 | -29.1 ± 0.3 |
| **AGT(V336D)** | 3.5 ± 0.2 | 17.2 ± 0.9 | 48.4 ± 1.0 | -31.2 ± 0.1 |
| **AGT(G170R)** | 3.8 ± 0.7 | 18.9 ± 1.0 | 49.8 ± 1.2 | -30.9 ± 0.5 |

The average stoichiometry calculated from all performed measurements is 1.02 ± 0.05.
