# Supplementary material for: Molecular Requirements for Peroxisomal Targeting of Alanine-Glyoxylate Aminotransferase as an Essential Determinant in Primary Hyperoxaluria Type 1
Source: PLoS Biol. 2012 Apr 17;10(4):e1001309. doi: 10.1371/journal.pbio.1001309 (PMC3328432; doi:10.1371/journal.pbio.1001309)
Supplement: Table S2 — Comparison of the structure of AGT and Pex5p. (DOC) [file pbio.1001309.s009.doc]

**Table S2**: Comparison of the structure of AGT and Pex5p

| **Structure-1** | **Structure-2** | **No. aligned residues** | **RMSD** | **P-score** |
| --- | --- | --- | --- | --- |
| **AGT-Pex5p complex: internal comparison** | | | | |
| AGT(A) | AGT(B) | 387 (100%) | 0.27 | 40.3 |
| Pex5p(A) | Pex5p(B) | 284 (99%) | 1.02 | 22.7 |
| AGT-Pex5p(A) | AGT-Pex5p(B) | 602 (89%) | 1.76 | 26.9 |
| **AGT-Pex5p complex *versus* apo AGT (1H0C)** | | | | |
| AGT(A) | AGT(1H0C) | 382 (100%) | 0.43 | 35.1 |
| AGT(B) | AGT(1H0C) | 382 (100%) | 0.38 | 35.8 |
| AGT_2_ | AGT(1H0C)_2_ | 766 (100%) | 0.41 | 66.9 |
| **AGT-Pex5p complex *versus* Pex5p in complex with SCP2 (2C0L)** | | | | |
| Pex5p(A) | Pex5p(2C0L) | 275 (100%) | 0.81 | 21.0 |
| Pex5p(B) | Pex5p(2C0L) | 273 (99%) | 1.14 | 18.3 |
| **AGT-Pex5p complex *versus* apo Pex5p (2C0M)** | | | | |
| Pex5p(A) | Pex5p(2C0M,A) | 268 (83%) | 1.85 | 9.3 |
| Pex5p(A) | Pex5p(2C0M,B) | 267 (85%) | 1.88 | 8.3 |
| Pex5p(A) | Pex5p(2C0M,C) | 267 (79%) | 1.90 | 7.3 |
| Pex5p(A) | Pex5p(2C0M,D) | 269 (84%) | 1.93 | 9.0 |
| Pex5p(B) | Pex5p(2C0M,A) | 264 (83%) | 2.10 | 5.2 |
| Pex5p(B) | Pex5p(2C0M,B) | 262 (83%) | 2.08 | 5.9 |
| Pex5p(B) | Pex5p(2C0M,C) | 262 (89%) | 2.04 | 7.6 |
| Pex5p(B) | Pex5p(2C0M,D) | 264 (88%) | 2.10 | 7.2 |
