# Supplementary material for: Molecular Requirements for Peroxisomal Targeting of Alanine-Glyoxylate Aminotransferase as an Essential Determinant in Primary Hyperoxaluria Type 1
Source: PLoS Biol. 2012 Apr 17;10(4):e1001309. doi: 10.1371/journal.pbio.1001309 (PMC3328432; doi:10.1371/journal.pbio.1001309)
Supplement: Table S3 — Estimated secondary structure content of purified AGT variants. (DOC) [file pbio.1001309.s010.doc]

**Table S3: Estimated secondary structure content of purified AGT variants.**

**Protein Helix [%] Strand [%] Turns [%] Unordered [%]**

wt 35 ± 3 19 ± 3 20 ± 2 25 ± 2

A328W 32 ± 1 18 ± 1 20 ± 0 29 ±1

Y330A 24 ± 10 22 ± 5 25 ± 2 29 ± 2

Y330W 36 ± 3 19 ± 1 19 ± 0 25 ± 3

G170R 38 ± 2 19 ± 7 18 ± 3 26 ± 7

V336D 31 ± 3 24 ± 3 20 ± 2 25 ± 5
