# Supplementary material for: Molecular Requirements for Peroxisomal Targeting of Alanine-Glyoxylate Aminotransferase as an Essential Determinant in Primary Hyperoxaluria Type 1
Source: PLoS Biol. 2012 Apr 17;10(4):e1001309. doi: 10.1371/journal.pbio.1001309 (PMC3328432; doi:10.1371/journal.pbio.1001309)
Supplement: Table S4 — Primer sequences used. (DOC) [file pbio.1001309.s011.doc]

**Table S4: Primer sequences used**

| **AGT variant** | **Forward primer** | **Reverse primer** |
| --- | --- | --- |
| wt | ggccatggcctctcac | gggtacctcacagcttcttctt |
| pEGFP | tcgaagatctatggcctctcacaagctg | cgtgaattctcacagcttcttcttggg |
| Y330W | gctgtacccgctggctgggactggagagacatcg | cgatgtctctccagtcccagccagcgggtacagc |
| Y330A | ggctgtacccgctggcgccgactggagagacatcg | cgatgtctctccagtcggcgccagcgggtacagcc |
| A328W | ccactgtggctgtaccctggggctatgactggagaga | tctctccagtcatagccccagggtacagccacagtgg |
| V376D | gaatgtggaccgcgacacggaggccctgag | ctcagggcctccgtgtcgcggtccacattc |
| L380D | cgtgacggaggccgacagggcggccctgc | gcagggccgccctgtcggcctccgtcacg |
| V376P | gaatgtggaccgcccgacggaggccctg | cagggcctccgtcgggcggtccacattc |
| L380P | gacggaggccccgagggcggccc | gggccgccctcggggcctccgtc |
| G170R | cccttgatggcttcagggaactctgccac | gtggcagagttccctgaagccatcaaggg |
| V336D | gactggagagacatcgacagctacgtcatagac | gtctatgacgtagctgtcgatgtctctccagtc |
| deltaKKKL | ctgcagcactgcccctagaagaagctgtgag | ctcacagcttcttctaggggcagtgctgcag |
