# Supplementary material for: Molecular Requirements for Peroxisomal Targeting of Alanine-Glyoxylate Aminotransferase as an Essential Determinant in Primary Hyperoxaluria Type 1
Source: PLoS Biol. 2012 Apr 17;10(4):e1001309. doi: 10.1371/journal.pbio.1001309 (PMC3328432; doi:10.1371/journal.pbio.1001309)
Supplement: Text S1 — Detailed description of AGT-Pex5p interface interactions. (DOC) [file pbio.1001309.s012.doc]

**SUPPORTING TEXT**

**Detailed description of AGT-Pex5p interface interactions**

As expected, the highest level of conserved interactions is observed at the C-terminus of the PTS1, where the terminal carboxylate of Leu392 and the preceding peptide bond Lys391-Leu392 are held by identical hydrogen bonds, involving Asn415, Asn526 and Asn561 of the Pex5p receptor (**Figures 2A, 4, S5**). As shown by the superposition of the two Pex5p(C)-AGT complexes (**Figure S6**), these interactions serve as the pivot of the bimolecular assembly. By contrast, none of the side chains of the three preceding residues (Lys391, Lys390, Lys389), apart from Van-der-Waals contacts with Pex5p(C), are involved in identical interactions when comparing the two AGT-Pex5p(C) complexes. Non-conserved side chain-mediated interactions by Asn393, Ser417 and Asn602 of the Pex5p receptor are only found in one of the two complexes, indicating significant conformational variability at the level of the AGT PTS1 segment.

Next to the PTS1 core interaction site, several residues, which are located in helix α13 and are thus part of the overall architecture of the C-terminal AGT domain, are involved in AGT–Pex5p(C) interactions (**Figures 2A and S5**). In particular, the side chain of Gln385 is hydrogen bonded to residues from TPR7 of the Pex5p receptor as well as the side chain of His572 in one of the two complexes.

Despite the observed differences in the AGT-Pex5p(C) arrangement (**Figure S6**), both of the bound AGT protomers interact with the C-terminal bundle domain of Pex5p(C), but by different specific interactions (**Figures 2A and S5)**. There is a hydrogen bond between Arg381 from AGT and Ser612 from Pex5p(C) in only one of the two complexes, whereas in the other complex a main chain carbonyl group from one of the interacting tip lobes of the AGT C-terminal domain is bound to Ser612. This interaction, in turn, replaces another hydrogen bond with the side chain of Arg608 from Pex5p, leaving the latter residue without any specific interactions in one of the two complexes. Moreover, the differences in the AGT-Pex5p(C) arrangement lead to a substantially different spacing of the Pex5p(C) TPR array in relation to AGT, generating an open and closed arrangement (**Figure S6**). As a consequence of this, in the AGT-Pex5p(C) complex that represents the closed arrangement, an additional interface, generated by the tips of TPR2 and TPR3 (**Figures 2B and S6**) and the AGT C-terminal domain, is observed. These additional interactions increase the overall area of the respective AGT-Pex5p(C) interface by around 120 Å^2^.

In addition, important intramolecular interactions can be observed within the C-terminal domain of AGT. The orientation of the PTS1 segment is fixed by hydrogen bonds with two residues of the loop connecting strand β8 and helix α12, involving the main chain carbonyl group of Gly329 and the side-chain hydroxyl group of Tyr330 **(Figure 2A)**. In contrast to most of the other interactions, this arrangement is identical in both AGT-Pex5p(C) complexes.
